# Acute Toxicity and Genotoxic Activity of Avocado Seed Extract (*Persea americana* Mill., c.v. *Hass*)

**DOI:** 10.1155/2013/245828

**Published:** 2013-11-05

**Authors:** Eduardo Padilla-Camberos, Moisés Martínez-Velázquez, José Miguel Flores-Fernández, Socorro Villanueva-Rodríguez

**Affiliations:** Centro de Investigación y Asistencia en Tecnología y Diseño del Estado de Jalisco, A.C., Avenida Normalistas 800, Colonia Colinas de la Normal, Guadalajara 44270, JAL, Mexico

## Abstract

The use of vegetal extracts requires toxicological and genotoxic evaluations to establish and verify safety before being added to human cosmetic, pharmaceutical medicine, or alimentary products. *Persea americana* seeds have been used in traditional medicine as treatment for several diseases. In this work, the ethanolic seed extract of *Persea americana *was evaluated with respect to its genotoxic potential through micronucleus assay in rodents. The frequency of micronuclei in groups of animals treated with avocado seed extract showed no differences compared to the negative control (vehicle); therefore, it is considered that the avocado seed extract showed no genotoxic activity in the micronucleus test.

## 1. Introduction

The fruit of *Persea americana*, commonly known as avocado, is an edible fruit from Central America which is easily adaptable in tropical regions [1]. The avocado has an olive-green peel and thick pale yellow pulp that is rich in fatty acids such as linoleic, oleic, palmitic, stearic, linolenic, capric, and myristic acids. This fruit is normally used for human consumption, but it also has been used as a medicinal plant in Mexico and elsewhere in the world [2].

The avocado seed represents 13–18% of the fruit, and it is a byproduct generally not utilized. Normally, the seed is discarded during the processing of the pulp. The seed waste may represent a severe ecological problem [3]. However, at the same time, it may be of interest to industry as a source of bioactive compounds. Its chemical composition is comprised of phytosterols, triterpenes, fatty acids, and two new glucosides of abscisic acid [4].

Several biological activities of the avocado seed have been reported such as antioxidant, antihypertensive, larvicidal, fungicidal, hypolipidemic, and recently amoebicidal and giardicidal activities [5–8]. Additionally, several studies have focused on the evaluation of acute toxicity of the fruit and leaves [9]. Avocado leaves showed cardiotoxic effects in mammals and birds [10–13]. Similarly, the mutagenicity of fruit and leaves extracts in human lymphocytes has been assessed [14]. However, no study has been done to examine the possible genotoxic activity of avocado seed extract. In this study, we evaluate the genotoxic effect of a *P. americana* seed extract *in vivo*, by induction of micronuclei in blood polychromatic erythrocytes of BALB/c mice.

## 2. Materials and Methods

### 2.1. Plant Material

Avocados were purchased from Michoacán, Mexico. The seeds were dried at 40°C and vacuum packaged until use.

### 2.2. Avocado Seed Extract

The *P. americana* seed extract is an ethanolic extract obtained through soxhlet reflux equipment and evaporated on a rotary evaporator as mentioned by Ramos et al. in 2004 [4]. Dried avocado seeds (1600 g) were ground to powder in a laboratory mill, defatted with petrol ether (55–75°C), and macerated with freshly distilled ethanol until exhaustion. After filtration, extracts were concentrated under vacuum at 35°C.

### 2.3. Animals

Forty-five, eight-week-old male BALB/c mice (23 ± 2 g) were purchased from the Zooterio of the University of Guadalajara. The mice were fed Standard Diet 20128 Tekland and water. They were kept at room temperature under a 12 hours of light and 12 hours of dark cycle at 22°C. Thirty mice were employed for acute toxicity test and fifteen mice for genotoxicity test. Animals were handled following the animal care guidelines in accordance with regulations enacted by the Federal Government of Mexico (NOM-062-ZOO-1999 and NOM-033-ZOO-1995).

### 2.4. Acute Toxicity Test

To determine the median lethal dose (LD_50_) of the *P. americana* seed extract, six groups of 5 mice each were administered one by one by oral gavage at different doses consisting of 125, 250, 500, 1000, and 2000 mg/kg using an orogastric tube (Popper). Mortality was recorded 24 hours after the administration of the extract. Animals were observed during one week to detect signs of delayed toxicity.

### 2.5. Genotoxicity Test

The genotoxicological study of the *P. americana* seed extract was carried out using an identification and quantification on the erythrocyte micronucleus formation test. According to the acute toxicity test results, the most appropriate extract dose for genotoxic study was selected. Three groups of 5 mice each were employed in the experiment. The first group was given *P. americana* seed extract at a dose of 250 mg/kg, dissolved in 1 : 1 alcohol-water solution; the second group was given colchicine dissolved in physiological saline at a dose of 4 mg/kg and was designated as the positive control, while the third group was assigned as the negative control and received the vehicle (1 : 1 alcohol-water solution), all solutions in an amount of 1 mL/kg. At 36 hours after administration of treatments, peripheral blood samples were collected by perforating the caudal vein, and drops were placed at the prestained slides with acridine orange as described by Hayashi and Sofuni, 1994 [15]. The micronucleated cells were scored under a fluorescence microscope. 1000 peripheral reticulocytes per mouse were analyzed, and the frequencies of micronucleated peripheral reticulocytes were scored in three slides per animal [16].

### 2.6. Statistical Analyses

LD_50_ value was determined through Probit analysis [17]. One-way ANOVA multicomparisons tests were used to identify any significant difference among genotoxicity tests between animal groups, and Fisher's least significant difference (LSD) was used to compare significant differences between groups. A *P* value < 0.05 was considered statistically significant. All data was analyzed using the software Statgraphics Version XVI.I.

## 3. Results

### 3.1. Acute Toxicity

**Figure 1:**
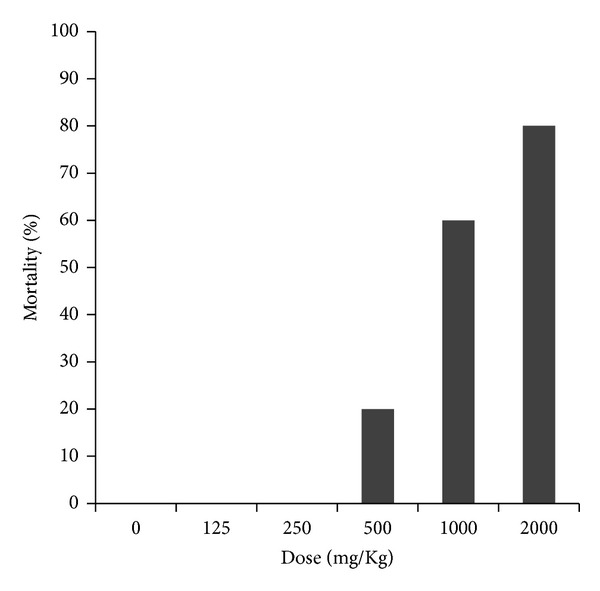
Percentage of mortality for determining the acute toxicity of the *P. americana *seed extract.

The *P. americana *seed extract administrated at doses of 500, 1000, and 2000 mg/Kg showed a mortality of 20, 60, and 80%, respectively. The animal groups treated with 125 and 250 mg/Kg, as well as the control group, showed no mortality (Figure 1). The LD_50_ calculated for the avocado seed extract was 1200.75 mg/kg. According to these results, a concentration of 250 mg/Kg was determined for genotoxicity testing.

### 3.2. Genotoxicity Test

The animal groups administered with 250 mg/Kg of *P. americana* seed extract and the negative control group showed a low amount of micronucleated cells, while the positive control administered with colchicine showed clear evidence of harm. There is no statistically significant difference between the group administered with the extract of *P. americana* seed and control group; however, there is a significant difference between both these groups in regard to the positive control (Table 1).

## 4. Discussion

The avocado (*P. americana*) is consumed by humans because of its organoleptic characteristics; furthermore, the pulp contains up to 33% oil, rich in monounsaturated fatty acids [3]. The avocado seed is discarded in the majority of countries, although in some countries such as Niger, it is consumed [18, 19]. This waste may represent an ecological or human contaminant.

In a study previously reported on acute and subacute toxicity of a *P. americana* aqueous extract, it was not possible to estimate the LD_50_ value with the doses tested (up to 10 g/kg). Also, in repeated doses, toxicity tests during 28 days showed no affectations in hematological and biochemical parameters. Therefore, the authors concluded that the aqueous extract appears safe at least on an acute and sub-acute basis [9]. We showed that the ethanolic extract of *P. americana* seed presents acute toxicity with a LD_50_ value of 1200.75 mg/kg. The acute toxicity differences found in the aqueous and ethanolic extracts may be due to chemical components obtained by different extraction methods used.

Micronucleus is an excellent genotoxic biomarker [15, 20]; therefore, the staining technique with acridine orange helps differentiate micronucleated cells. The evaluation of micronucleus frequencies *in vivo* is one of the primary genotoxicity tests recommended internationally by regulatory agencies for product safety assessment [21]. Based on the international working group that evaluates the micronucleus test, this should be used when no signs of toxicity at maximal possible concentration are seen [22, 23]. Accordingly, from the acute toxicity test, the dose of 250 mg/kg was selected for the genotoxicity test [24].

This is the first study on the genotoxicity of the ethanolic avocado seed extract. The micronucleus induction with the *P. americana* seed extract showed no statistical difference with regard to the negative control, but with regard to the positive control it did. Therefore, it is considered that the *P. americana* seed extract showed no genotoxic activity with the micronucleus test. There is a study that demonstrates the genotoxicity of the avocado fruit and leaf extracts in human peripheral lymphocytes [14]; however, this study was carried out *in vitro*. There are different toxic effects *in vitro* and *in vivo*, especially when it is administered orally.

Reports of genotoxicity have revealed that many plants used as food or in traditional medicine have cytotoxic, mutagenic, and genotoxic properties [25]. This indicates the need to understand the toxicological profile of substances that are in direct or indirect contact with humans.

To complement the toxicological profile of the avocado seed extract, it is also necessary to test for other areas of potential damage, such as those related to the immune system and those that alter endocrine function.

## 5. Conclusion

The ethanolic extract of the *P. americana* seed showed an acute toxic effect at concentration starting at 500 mg/kg. *In vivo* mutagenicity on peripheral blood cells of the seed extract was not observed. However, this study needs to be supported with experimental toxicity studies using isolated compounds. The lack of *in vivo* genotoxic activity of the extract allows us to hope that the *P. americana* seed extract could be used as a possible food, cosmetic, or pharmaceutical material.

## Figures and Tables

**Table 1:** Micronucleated peripheral reticulocytes (MNRET) formed by *P. americana seed* extract.

Samples	Dose (mg/kg)	MNRET/1000 RET	
		Number	%
Alcohol-water solution		10.6 ± 1.1	1.06 ± 0.11
*P. americana seed *extract	250	16.4 ± 4.5	1.64 ± 0.45
Colchicine	4	189.2 ± 19.3	18.92 ± 1.93*

The data are presented as mean standard deviation of three repetitions. Asterisks denote statistical differences compared with control (*P < 0.05).
